# In Vivo Evidence on the Emerging Potential of Non-Digestible Oligosaccharides as Therapeutic Agents in Bacterial and Viral Infections

**DOI:** 10.3390/nu17061068

**Published:** 2025-03-19

**Authors:** Amirmohammad Afsharnia, Yang Cai, Arjen Nauta, Andre Groeneveld, Gert Folkerts, Marc M. S. M. Wösten, Saskia Braber

**Affiliations:** 1 Division of Pharmacology, Utrecht Institute for Pharmaceutical Sciences, Faculty of Science, Utrecht University, 3584 CB Utrecht, The Netherlands; a.afsharnia@uu.nl (A.A.); g.folkerts@uu.nl (G.F.); 2 Department of Pharmacology, Jiangsu Provincial Key Laboratory of Critical Care Medicine, School of Medicine, Southeast University, Nanjing 210009, China; cai_yang@seu.edu.cn; 3 FrieslandCampina, 3818 LE Amersfoort, The Netherlands; arjen.nauta@frieslandcampina.com (A.N.); andre.groeneveld@frieslandcampina.com (A.G.); 4 Division of Infectious Diseases and Immunology, Department of Biomolecular Health Sciences, Faculty of Veterinary Medicine, Utrecht University, 3584 CL Utrecht, The Netherlands; m.wosten@uu.nl

**Keywords:** non-digestible oligosaccharides, human milk oligosaccharides, prebiotics, Bacterial infections, viral infections, pathogens, gut microbiota, immune response, barrier function, short-chain fatty acids

## Abstract

The issue of antibiotic-resistant bacterial infections, coupled with the rise in viral pandemics and the slow development of new antibacterial and antiviral treatments, underscores the critical need for novel strategies to mitigate the spread of drug-resistant pathogens, enhance the efficacy of existing therapies, and accelerate the discovery and deployment of innovative antimicrobial and antiviral solutions. One promising approach to address these challenges is the dietary supplementation of non-digestible oligosaccharides (NDOs). NDOs, including human milk oligosaccharides (HMOs), play a vital role in shaping and sustaining a healthy gut microbiota. Beyond stimulating the growth and activity of beneficial gut bacteria, NDOs can also interact directly with pathogenic bacteria and viruses. Their antiviral and antibacterial properties arise from their unique interactions with pathogens and their ability to modulate the host’s immune system. NDOs can function as decoy receptors, inhibit pathogen growth, bind to bacterial toxins, stimulate the host immune response, exhibit anti-biofilm properties, and enhance barrier protection. However, a notable gap exists in the comprehensive assessment of in vivo and clinical data on this topic. This review aims to provide an in-depth overview of the in vivo evidence related to the antiviral and antibacterial effects of various NDOs and HMOs, with a focus on discussing their possible mechanisms of action.

## 1. Introduction

Considering the prevalence of viral pandemics in the last decade, antibiotic-resistant bacterial infections, and the reduced pace of developing antibacterial and viral drugs, there is an urgent need to uncover innovative strategies, such as non-digestible oligosaccharide (NDO) supplementation to the diet, to fight pathogens [1,2].

NDOs, a type of dietary fiber, represent a class of carbohydrates that resist gastrointestinal digestion [3]. NDOs have the capability to reach the large intestine in an almost intact form, where their utilization by gut bacteria contributes to various health benefits [4,5,6,7]. The first readily available natural source of NDOs for infants is breast milk, which contains a variety of human milk oligosaccharides (HMOs) [8]. Human milk contains significantly more oligosaccharides than non-human mammalian milk, with the highest number of identified, characterized, and quantified oligosaccharides. It has 10–15 g/L of HMOs, 100 to 1000 times higher than bovine milk, and 10 to 100 times greater than in other mammals (like goats and sheep), though genetic factors and lactation stages complicate cross-species comparisons [9].

NDOs can be naturally found in milk, honey, fruits, vegetables, whole grains, and nuts as well as other sources [10]. NDOs can also be extracted from other natural sources like chitosan oligosaccharides (COSs) from crustacean shells and mannan oligosaccharides (MOSs) from yeast cell walls [11,12]. However, they have to be obtained by the chemical or enzymatic degradation of non-digestible polysaccharide contents [13]. In addition, certain NDOs can be synthesized through food processing techniques and incorporated into food products for various applications [6,7].

Oligosaccharides can be classified into distinct categories based on their chemical composition [13,14]. Structurally, NDOs contain a diverse array of less than 20 monomeric building blocks such as fructo-oligosaccharides (FOSs), galacto-oligosaccharides (GOSs), or MOSs [15]. More than 200 HMOs have been identified that are composed of five monosaccharide residues: glucose (Glc), galactose (Gal), *N*-acetylglucosamine (GlcNAc), fucose (Fuc), and sialic acid, which are linked by various glycosidic bounds to generate linear and branched structures [16]. Lactose often serves as the core structure in HMOs. The synthesis of HMOs is gaining importance, particularly in infant nutrition and healthcare.

Both NDOs and HMOs have been shown to play a significant role in shaping and preserving the healthy gut microbiota composition [17]. The fermentation of NDOs and HMOs by gut bacteria leads to the production of short-chain fatty acids (SCFAs) such as butyrate, acetate, and propionate [18]. NDOs and HMOs, along with the SCFAs they promote, play important roles in maintaining gastrointestinal tract homeostasis by their anti-inflammatory, antipathogenic, and immunoregulatory properties [4,5,8,19,20].

**Figure 1:**
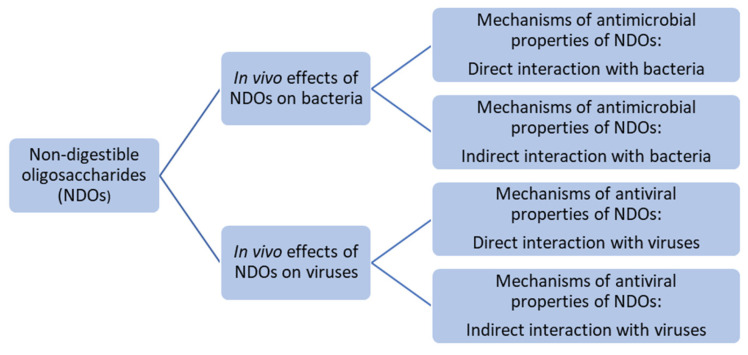
Schematic representation of the topics covered in this review.

Besides stimulating the activation and presence of beneficial bacteria in the gut by NDOs, NDOs also have the capacity to interact with pathogenic bacteria and viruses [19,21,22,23,24,25,26,27,28,29]. A previous review examined the literature on the in vitro antipathogenic properties of NDOs and HMOs [30]. The use of NDOs and HMOs as antiviral and antibacterial agents originates from their unique properties and the diverse ways in which they interact with the host’s immune system and/or pathogens. For example, HMOs and NDOs can directly interfere with pathogens as decoy receptors, inhibit their growth, bind to bacterial toxins, stimulate the host immune system, exhibit anti-biofilm properties, show barrier-protective effects, and boost the growth of beneficial gut bacteria [30]. Despite all of the promising in vitro antipathogenic findings of NDOs and HMOs, available in vivo and clinical data on this topic are scarce. Therefore, this review aimed to provide a comprehensive overview of the in vivo findings related to the antiviral and antibacterial effects of a wide range of NDOs and HMOs as well as discuss their possible mechanism of action (Figure 1).

## 2. Antimicrobial Effect of NDOs

### 2.1. In Vivo Evidence for Antimicrobial and Anti-Infective Effects of NDOs

There is growing interest in the human and animal health benefits derived from the antimicrobial effects of NDOs. Breast milk is the recommended source of nutrition for newborns and has a unique composition of naturally occurring ingredients that aim to support development and health during infancy. In addition, breast milk contains high levels of bioactive components, such as HMOs (5 to 25 g/L in human milk) [31], which promote a healthy microbiome and immune system to protect against pathogenic microbes [32,33]. Breastfeeding can reduce the incidence of diarrhea, pneumonia, otitis media, and atopic dermatitis in infants and children [34,35,36], which may be due to the anti-biofilm and anti-microbial activity of HMOs, as confirmed in vitro [37]. An in vivo study confirmed that an HMO mixture isolated from breast milk could reduce the burden of *S. agalactiae* without disturbing the vaginal microbiota in a murine *S. agalactiae* vaginal colonization model [38]. Moreover, fucosyloligosaccharides of human milk have the capacity to inhibit the colonization of *Campylobacter jejuni* in mice [39]. 3′-Sialyllactose (3′SL) sodium salt, a sialylated HMO, cleared the *Helicobacter pylori* colonization in rhesus monkeys [40]. However, the HMO, 3′SL, did not inhibit *H. pylori* colonization in human clinical trials [41]. Intratracheal administration of HMOs, lacto-N-neotetraose (LNnT) and sialylated LNnT, reduced the pneumococcal burden in the lungs and protected against bacteremia, inhibiting pneumococcal pneumonia in rabbits [42]. Consistent with this study, intranasal administration of GOSs, which are structural mimics of HMOs, can reduce naturally occurring respiratory infections in calves, as observed by a decrease in *Pasteurellaceae* CFUs in bronchoalveolar lavage fluid [43]. The anti-infective and beneficial effects of GOS have been found in different experimental animal models including *Salmonella Typhimurium*-infected mice [44], *Listeria monocytogenes*-infected mice and guinea pigs [45,46], *Edwardsiella tarda*-infected rockfish [47], and in calves with naturally occurring lung infections [48]. Sialyllactose and pectic oligosaccharides can improve the bacterial clearance of *Pseudomonas aeruginosa* lung infection in mice [49,50], while FOS supplementation through drinking water reduced the shedding of *S. Typhimurium* in pigs [51]. Dietary FOSs alleviated the severity of naturally occurring respiratory infections in calves, as evidenced by a lower proportion of moderate/severe lung lesions and anti-inflammatory properties [52]. Furthermore, dietary inulin, FOS, mannan oligosaccharides (MOSs), and lignin alleviated intestinal infections and inflammatory intestinal damage in *Salmonella*-challenged chickens [53,54,55,56,57]. FOS feeding also increased ROS production and the bacterial killing of *P. aeruginosa* in a murine antibiotic-induced lung defense impairment model [58]. Moreover, alginate-chitosan oligosaccharide-based hydrogels are promising treatments for infections with *Staphylococcus aureus*, *Escherichia coli*, *Candida albicans*, and *Bacillus subtilis* and accelerated wound healing in mice and rats [59,60].

In conclusion, a growing number of in vivo studies have highlighted the antimicrobial and anti-infective benefits of NDOs. Table 1 summarizes the evidence supporting their in vivo antimicrobial efficacy.

### 2.2. Mechanisms of Antimicrobial Properties of NDOs: Direct Interaction with Pathogens

Despite the anti-infective effects of NDOs found in different experimental animals as described above, knowledge of the mechanisms remains limited. Currently, many studies still focus on characterizing the gut microbiota-dependent mechanisms of NDOs in inflammatory diseases [61,62,63,64]. Notably, as an alternative to antibiotics, NDOs contain powerful biological activities and may have unique mechanisms of gut microbiota-independent action including anti-biofilm activity, influence on cell recognition of pathogens, interference with signal transduction, inhibition of α-glucosidase and lipase, and disruption of cell wall/membrane biosynthesis [30,65]. Although some of these mechanisms of action have been discovered and studied in vitro, these studies could contribute to a better understanding of the antimicrobial activity of NDOs in vivo.

#### 2.2.1. NDOs Decrease Bacterial Biofilm Formation and Activity

Townsend and his colleagues reported that an HMO mixture (purified from breast milk) showed anti-biofilm activity against *Streptococcus agalactiae* and *S. aureus* and exhibited a growth inhibition of *S. agalactiae* and *Acinetobacter baumannii* in vitro [66]. High-resolution scanning electron microscopy and confocal laser scanning microscopy showed that these HMOs purified from breast milk could alter the *S. agalactiae* morphology, causing a truncated chain phenotype and a condensed packing morphology within the in vitro biofilm [66,67]. These HMOs may limit the growth space of the biofilm by positioning on top of the *S. agalactiae* biofilm [37,67]. The adhesion and virulence of most pathogenic bacteria are dependent on biofilm formation, therefore, the inhibition of biofilms by HMOs reduces pathogenic virulence and thus inhibits infection [68]. About 200 unique HMOs have been identified in human breast milk, but so far, no single HMO has been found to be as effective as heterogeneous HMO extracts against the biofilm formation of *S. agalactiae* in vitro, as investigated by Craft and Townsend [37]. This suggests that there may be unknown synergistic effects on the inhibition of biofilm formation between single entity HMOs, as also indicated by Craft and Townsend [37]. Several commercial oligosaccharides have also been shown to inhibit the biofilm formation of pathogenic bacteria. Alginate oligosaccharides (AOSs) and chitosan oligosaccharides (COSs) prevent *S. aureus* biofilm formation and enhance the inhibitory effect of clindamycin on *S. aureus* biofilm formation in vitro [69]. Moreover, inulin stimulates the biofilm formation of *P. aeruginosa*, whereas its hydrolyzed form, FOS, dramatically reduces biofilm formation in vitro [70]. Different structures of oligosaccharides may have different effects on bacterial biofilms. The fine structure of oligosaccharides (e.g., specific chain length, specific oligosaccharide sequences, and charge profile) may modify and affect the intrinsic stability of biofilms including altering the growth rate and mechanical properties of biofilms [71].

#### 2.2.2. NDOs Affect Interactions Between Host Cells and Bacteria: Inhibition of Recognition and Adhesion

Effective bacterial adhesion facilitates the escape of pathogens from the natural cleansing action of the host and eventually contributes to their colonization and invasion including the formation of biofilms [72]. Microorganisms such as bacteria use lectins (a carbohydrate-binding protein) to interact specifically with glycans on host cells [73,74]. *P. aeruginosa* is a representative example of the use of lectins to adhere to host cells. LecA from *P. aeruginosa* is a lectin that specifically targets galactose and is essential for bacterial internalization into the host cell [75]. LecB from *P. aeruginosa* strongly binds fucose and fucose-containing oligosaccharides and contributes to bacterial adhesion to airway epithelial cells [76]. Thus, various, NDOs (e.g., 2′-fucosyllactose, 3-fucosyllactose, and GOSs) may function as decoys, capturing *P. aeruginosa* through interactions with lectins and reducing the bacterial adhesion to host cells [77,78]. NDOs (e.g., pectic oligosaccharides, sialyllactose) have been shown to clear *P. aeruginosa* in murine infection models, which may be attributed to their competitive binding to host cells with bacterial lectins or to the improved immune function induced by the altered gut microbiota composition [49,50,79]. The most common cause of urinary tract infections is uropathogenic *E. coli* adhesion to α-mannopyranosyl ligands on the surface of host uroepithelial cells, and the adhesin involved is FimH, which contains a mannose-specific lectin structural domain [80]. Thus, mannose and extracts rich in mannose, such as mannan oligosaccharides, can be used as an antagonist of FimH-mediated bacterial adhesion in vitro and in vivo [81,82,83].

The anti-adhesive properties of different NDOs, including bovine milk oligosaccharides, HMOs, 2′-fucosyllactose, 6′-sialyllactose, GOSs, FOSs, AOSs, and pectic oligosaccharides, against different pathogens continue to be discovered [84,85,86,87,88,89,90,91], but these findings need to be confirmed in future in vivo studies.

The *S. aureus*, *A. baumannii*, *P. aeruginosa*, and *Enterobacter* species belong to notorious ESKAPE pathogens, aptly named for their ability to “evade” the action of antimicrobials. The mechanism of NDOs that interferes with carbohydrate-protein recognition is designed to prevent pathogen colonization and thus potentially decrease biofilm formation. This NDO-induced interference with colonization is not aimed at killing the invading pathogen, and therefore does not generate the selective pressure that leads to drug resistance [80,92]. A large number of mimetic and neoglycan-adhesive structures (e.g., octopus glycosides, glycodendrimer, multivalent fucosides, butyl α-D-mannoside) mimic the carbohydrate structures and functions of NDOs and HMOs. These compounds act as antagonists of carbohydrate–protein interactions involved in bacterial infections, exerting anti-adhesive properties, which have been discussed in several comprehensive reviews [65,80,93,94,95,96,97].

#### 2.2.3. NDOs Increase the Permeability of Bacterial Cell Membranes and the Efficacy of Antimicrobial Drugs

The combined use of NDOs and antimicrobial drugs has gained increasing attention [98]. Oligosaccharides may enhance the effectiveness of antimicrobial agents by sensitizing pathogenic bacteria. HMO mixtures from donor breast milk enhanced the antimicrobial activity of four classes of intracellularly targeted antibiotics: aminoglycosides, lincosamides, macrolides, and tetracyclines. In contrast, these HMO mixtures did not enhance the antibacterial activity of cell wall-targeted antibiotics including β-lactams, cephalosporins, carbapenems, and glycopeptides [37,99]. The chitosan-based oligosaccharide compound AVR-25 in combination with the antibiotic imipenem protected young (10–12 weeks old) and old (16–18 months old) mice from multiple microbial infections after cecal ligation and puncture [100]. Chitin oligosaccharides can reduce the antibiotic dose and antibiotic-induced side effects in patients after surgery [101]. COSs and clindamycin have synergistic inhibitory effects on *S. aureus* biofilm formation, whereas AOSs can enhance *S. agalactiae* susceptibility to trimethoprim in vitro [69]. In addition, GOSs enhance the antibacterial activity of tetracyclines (doxycycline, oxytetracycline) and macrolides (tilmicosin) against the bovine respiratory pathogen, *M. haemolytica*, by increasing membrane permeability [43]. These antibiotics inhibit bacterial protein synthesis by crossing the bacterial membrane and binding to ribosomal subunits [99]. Therefore, it is hypothesized that the altered membrane permeability induced by NDOs may increase the access of antibiotics to the ribosome, thereby enhancing the antimicrobial activity of the antibiotics. An increase in membrane permeability induced by NDOs could result from multiple mechanisms. NDOs can interfere with the bacterial outer membrane, potentially disrupting nutrient flow across the membrane, which impedes bacterial survival [102]. Moreover, the presence of NDOs may lead to imbalances in intra- and extracellular ion concentrations or alter charge distributions, further exacerbating membrane instability and damage [103,104]. The bacterial outer membrane is particularly susceptible to disruption by NDOs, and this disruption can lead to the leakage of vital cellular contents, accelerating the death of pathogenic bacteria. For example, COSs and AOSs have the ability to damage bacterial cell membranes, supporting the idea that NDOs can destabilize bacterial membranes and contribute to bacterial death [105,106]. He and colleagues reported that COSs synergize with azithromycin in inhibiting the growth of wild-type and drug-resistant *P. aeruginosa* in vitro [105]. AOSs have been reported to increase the efficacy of macrolide, β-lactam, and tetracycline antimicrobial drugs against different *Pseudomonas*, *Acinetobacter*, and *Burkholderia* genotypes in vitro [106]. Unfortunately, most of these mechanisms have been studied in vitro, while more in vivo experiments are needed to demonstrate the additional or synergistic effects of NDOs when combined with antimicrobial drugs. In summary, NDOs can mediate bacterial growth inhibition through different mechanisms. These mechanisms include the NDO-mediated (1) inhibition of bacterial biofilms; (2) decreased ability of bacteria to adhere to host cells; and (3) increased permeability of bacterial cell membranes.

### 2.3. Mechanisms of Antimicrobial Properties of NDOs: Indirect Interaction with Pathogens

#### Effects of NDOs on Microbiota Composition, SCFA Production, Barrier, and Immune Function During Bacterial Infections

It is well-known that the gut microbiota plays an important role in the prevention of infection and immunity. The gut microbiota integrates environmental signals (e.g., diet type, antibiotic use) with genetic and immune signals to influence host metabolism, immunity, and infection response [107]. The gut microbiota plays a key role in shaping the innate immune system [107], and during infection, pathogens compete with commensal bacteria for resources, disrupting the immune–microbiome crosstalk. Customized diets can alter the microbiota, enhancing immune responses to infections [108]. For example, a diet rich in NDOs promotes bacterial fermentation to increase the SCFA levels, which will boost immunity to eliminate pathogen threats [109].

Recent studies are beginning to reveal a link between dietary microbiota regulation and bacterial infections. The Western diet profoundly affects the structure of the gut microbiome and adversely affects host immunity [110]. For example, a high-fat diet can alter the composition of the gut microbiota and induce transient mucosal immunosuppression, leading to a higher susceptibility to *Salmonella enterica* serovar Typhimurium and *Listeria monocytogenes* infections [111], whereas switching to a diet with NDO-rich dietary fiber (30% inulin) improved the gut microbiota composition and restored mucosal and systemic CD4 T cell function and immunity in humans and mice [112]. HMOs are the most important regulators of gut microbiota development in infants and have been shown to promote an increased abundance of *Bifidobacterium* spp. and *Lactobacillus* spp. [113]. Recurrent respiratory infections in children have been associated with an imbalance in the gut microbiota, as evidenced by a significant decrease in the number of bifidobacteria and lactobacilli and an increase in the number of *E. coli*. Interestingly, a probiotic cocktail therapy (containing *Bifidobacterium infantis*, *Lactobacillus acidophilus*, *Enterococcus faecalis*, and *Bacillus cereus*) increased the bifidobacteria and lactobacilli counts and restored the intestinal microecological balance in pediatric patients, resulting in reduced infection symptoms and antibiotic use [114]. In addition, increasing clinical studies have demonstrated that HMOs (e.g., 2′-FL, LNnT) may enhance the immune response against respiratory infections by remodeling the homeostasis of the gut microbiota (mainly by increasing the abundance of bifidobacteria and the concentration of microbiota metabolite SCFAs) [115,116,117,118,119,120].

Infants and young children who are not breastfed are at higher risk of developing infections [34,121]. Supplementation with short and long chain inulin in the first year of life beneficially modulates the gut microbiota, resulting in higher levels of bifidobacteria in the first 6 months of life, which is associated with a shorter duration of spontaneous infection [122]. Acidic oligosaccharides from pectin (pAOSs) have been shown to improve *Pseudomonas aeruginosa*-induced lung infections by modulating the gut microbiota, inducing a shift in the Th2/Th1 immune balance to a Th1 response and inducing M1 macrophage activation in mice. This has been partially explained by the fact that pAOSs stimulate the growth of bifidobacteria and the production of SCFAs, which can shift Th2 responses to Th1 responses and promote Treg activity [49]. In addition, dietary COSs may attenuate enterotoxigenic *E. coli*-induced intestinal inflammation in piglets by increasing the diversity of the gut microbiota and the abundance of *Bacteroidetes* as well as by restoring the Th17/Treg immune balance [123]. In *Salmonella Enteritidis*-infected young chickens, MOSs and xylooligosaccharides (XOSs) altered the relative abundance of specific microbiota and the immune response during infection, and these positive effects were associated with reduced *S. entericaus* colonization capacity [124]. Among them, chickens fed XOSs were enriched in the genera *Lactobacillus*, *Roseburia*, and *Clostridium*, whereas *Ruminococcus*, *Coprococcus*, and *Enterococcus* species were increased in the MOS-treated group [124]. In addition, XOSs were shown to prevent *Salmonella* infection in mice by increasing the bifidobacterial abundance and by decreasing *Salmonella* colonization through maintaining the level of intestinal SCFAs and inhibiting their bacterial adhesion ability [125]. Similarly, GOSs have been shown to protect mice and chickens from *Salmonella* and *E. coli* infections by inducing changes in the gut microbiota composition (primarily an increase in abundance of beneficial bacteria) and increasing the production of SCFAs [126,127]. Moreover, GOSs and polidextrose enriched formula protect infants against respiratory infections by increasing intestinal Bifidobacteria and *Clostridium* cluster I colonization [128]. In turn, a high sucrose diet impaired gut microbiota homeostasis in mice, and induced lower levels of SCFAs and branched-chain fatty acids (BCFAs), promoting susceptibility to *S. Typhimurium* infection [129].

Enrichment of beneficial bacteria induced by NDOs in the gut favors the balance of the intestinal microenvironment including an enhancement of barrier function and mucus secretion, reduction in pathogen colonization, and the inhibition of pro-inflammatory mediator release. MOS supplementation has been reported to enhance intestinal immune barrier function and attenuate inflammatory responses in *Aeromonas hydrophila*-infected fishes [130,131]. In addition, dietary supplementation with MOS-selenium can improve the intestinal mucosal barrier, regulate the composition of gut microbiota, and prevent enterotoxigenic *E. coli*-induced diarrhea in weaned piglets [132]. GOSs reduced the colonization of *E. coli* O157 in mice and alleviated subsequent inflammation by enhancing the intestinal barrier function. In addition, GOSs promoted the growth of beneficial bacteria and increased the SCFA levels in the intestine, which may also be one of the mechanisms for alleviating *E. coli*-induced inflammation in these mice [126]. NDO-induced growth of beneficial bacteria and the subsequent production of SCFAs and antimicrobial peptides may contribute to the maintenance of immune homeostasis, protection of the integrity of the intestinal epithelium, and the suppression of proinflammatory responses in the intestinal tract during infections [126,133,134,135].

**Figure 2:**
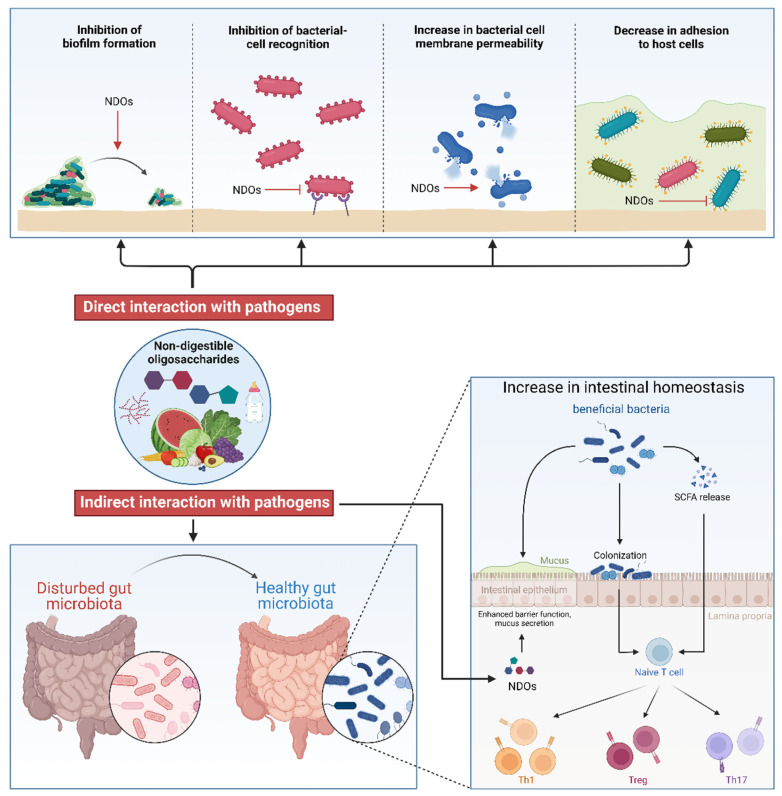
Hypothesis for the mechanism of action of non-digestible oligosaccharides (NDOs) against bacterial infections. This illustration highlights the principal mechanisms underlying the antibacterial action of NDOs via both direct and indirect interactions with pathogenic bacteria. As a potential alternative to antibiotics, NDOs demonstrate potent bioactivities and may employ unique mechanisms of action. These include directly altering the physiological properties of bacteria, such as exhibiting anti-biofilm activity [66,67,69,70], modifying pathogen recognition by host cells [30,65], increasing bacterial cell membrane permeability [43,69,99,101,104,105,106], and inhibiting pathogen adhesion to host cells [77,81,82,83,84,85,86,87,88,89,90,91]. Furthermore, NDOs have the capacity to directly stimulate epithelial cells and immune cells, enhancing epithelial barrier function and intestinal immunity to counteract bacterial adhesion and invasion [43,52,136,137,138,139]. Notably, the regulation of gut homeostasis by NDOs is vital in the context of infection and immunity. The gut microbiota combines environmental signals, such as dietary NDOs, with genetic and immune signals to influence host metabolism, immunity, and the response to infections [117,118,119,123,124,125,126,127,132]. By stimulating the growth of beneficial bacteria and increasing the SCFA levels, NDOs promote immune system activation and maintain homeostasis within the intestinal microenvironment. This includes reinforcing barrier function, increasing mucus secretion, reducing pathogen colonization, and inhibiting the release of pro-inflammatory mediators. Created with BioRender.com (accessed on 27 August 2024). NDOs, non-digestible oligosaccharides; SCFA, short-chain fatty acid; Th1, T-helper 1 cells; Treg, regulatory T cells; Th17, T-helper 17 cells.

Notably, NDOs also directly modulate epithelial cell function. NDOs, such as FOSs, GOSs, AOSs, COSs, MOSs, and XOSs, protect the epithelial barrier function via their prebiotic activities in both in vivo and in vitro models, as summarized in our previous review [136]. These NDOs interact with cell surface receptors, including toll-like receptors (TLRs), calcium-sensing receptors, and mannose receptors, to positively regulate tight junctions [136]. The greater density of tight junctions leads to improved barrier function, effectively hindering pathogen adhesion and invasion, which was proven in *M. haemolytica*-infected airway epithelial cells with NDO interventions [43,52,96]. In addition, NDOs can promote intestinal immunity through direct effects on the cells of the intestinal immune system including dendritic cells, macrophages, and mast cells [137,138]. NDOs bind to target receptors on immune cells, including TLRs, carbohydrate-binding domains, and peroxisome proliferator-activated receptor γ, facilitating the suppression of pro-inflammatory mediators and the release of anti-inflammatory mediators [139]. Certainly, further exploration is warranted across a broader spectrum of infectious diseases. Overall, NDOs can promote the establishment and maintenance of gut homeostasis, including an increased abundance of beneficial bacteria and subsequent beneficial fermentation products, like SCFAs, facilitating intestinal development, enhancing barrier function, and stimulating immune maturation to protect the host from pathogenic bacteria. The proposed mechanisms by which NDOs combat bacterial infections are illustrated in Figure 2.

**Table 1:** In vivo evidence for the antimicrobial effects of NDOs.

Treatment Target	NDOs (Amount, Application, etc.)	Model Description (Pathogen, Procedure, etc.)	Effects	References
Infants	HMOs (2′FL + LNnT; 1.0 g/L + 0.5 g/L)	Naturally acquired infections	Reduced risk of respiratory infections Decreased use of antibiotics or antipyretics Increased abundance of intestinal bifidobacteria Increased levels of SCFAs	[115,116,117,118,119,120]
	Inulin-type oligosaccharides (scFOS + lcFOS; 4 g/L + 4 g/L; 50:50 ratio ± 10% each)		Decreased duration of infection Higher percentage of bifidobacteria	[122]
	GOS/PDX formula (GOS + PDX: 4 g/L + 4 g/L; 50:50 ratio)		Reduced incidence of respiratory infections Increased abundance of intestinal bifidobacteria and *Clostridium* cluster I colonization	[128]
Adults	Oral 10 g or 20 g/day 3′SL	A positive screening test for *H. pylori* infection	Did not inhibit *H. pylori* colonization	[41]
BALB/c mice	100 μL PBS containing 2 mg neutral HMOs	*C. jejuni* 287ip orally	Inhibited colonization of *C. jejuni*	[39]
	5% pAOS extracted from citrus	*P. aeruginosa* strain PAO1 via airway administration	Increased growth of *Bifidobacterium* species, *Sutturella wadsworthia*, and *Clostridium* cluster XIVa Increased production of butyrate and propionate Promoted Th1 polarization Recruited polynuclear leukocytes and macrophages Stimulated M1 macrophage activation and IL-10 release Decreased TNF-α release Increased bacterial clearance	[49]
	2 g GOS/kg BW	*E. coli* O157 (ATCC35150) via intragastric administration	Reduced colonization of *E. coli* Enhanced the gut barrier function Relieved *E. coli*-induced inflammation Promoted the growth of beneficial bacteria such as *Akkermansia*, *Ruminococcaceae*, and *Bacteroides* Improving SCFA levels in the intestine	[126]
	Oral 200, 1000, and 2000 mg/kg 3′SL or 6′SL of BW	*P. aeruginosa* K via intranasal inoculation	Enhanced bacterial clearance in *P. aeruginosa* K-infected mice	[50]
C57BL/6 mice	1 mg (10 μL of 100 mg/mL) purified HMOs or LNT	*S. agalactiae* via vaginal colonization	Reduced *S. agalactiae* vaginal burdens	[38]
	5% XOS	*Salmonella Typhimurium* orally	Reduced *Salmonella* counts Stimulated *Bifidobacterium animalis* growth Suppressed the *Salmonella*-induced inflammation	[125]
SLC: ICR mice	2.5 mg GOS/100 μL sterile PBS by transmural injection	*S. Typhimurium SL1344nalr* by transmural injection	Prevented the adherence or invasion of *S. Typhimurium* to enterocytes Reduced pathological damage	[44]
Guinea pigs	100 g/kg GOS or XOS	*L. monocytogenes* orally	Improved the resistance of guinea pigs to *L. monocytogenes*	[46]
Rabbits	Intratracheal 20 nM LNnT or LSTc/0.2 mL saline	*Pneumococcal pneumonia* intratracheally	Reduced pneumococcal burden in the lungs	[42]
Calves	Intranasal 1.5 g GOS/10 mL saline	Naturally acquired infections	Lowered amount of *Pasteurellaceae* CFUs in bronchoalveolar lavage fluid Reduced naturally acquired respiratory infections	[43]
	Calf milk replacer with 1% or 2% GOS		Suppressed both local and systemic inflammation Reduced the *M. haemolytica* positivity Inhibit NLRP3 inflammasome activation in the lungs	[48]
	Calf milk replacer with 0.25% FOS		Decreased macrophage numbers in BALF Decreased IL-8, IL-6, and IL-1β concentrations in BALF and blood Decreased severity of lung lesions	[52]
Piglets	500 mg COS/kg BW	Enterotoxigenic *E. coli* orally administered for 3 consecutive days of the experiment	Alleviated the symptoms associated with the infection Lowered the abundance of intestinal *Lactobacillus*, *Streptococcus*, and *Anarovovrio* Increased the level of *Muribaculaceae_unclassified* and *Prevotella* Inhibited the expression of STAT3 mRNA Regulated Th17/Treg balance-related immune signaling	[123]
	0.4 mg/kg MOS-selenium supplemented diet	Enterotoxigenic *E. coli* orally administered once per week	Increased average daily gain and average daily feed intake Decreased diarrhea index and frequency Decreased the proportion of lipopolysaccharide biosynthesis in ileal microbial community Regulated colonic microbiota community composition Decreased inflammatory stress and oxidative stress Ameliorated intestinal mucosa barrier	[132]
Chickens	2 g XOS/kg BW	*S. Enteritidis* orally	Decreased cecal *Salmonella Enteritidis* counts Increased genera *Lactobacillus*, *Roseburia* and *Clostridium* Reduced expression of IL-6 and TNF-α	[124]
	1 g MOS/kg BW		Decreased cecal *Salmonella Enteritidis* counts Increased *Ruminococcus*, *Coprococcus* and *Enterococcus* species Reduced expression of IL-6, TNF-α, and INF-γ	
	1% functional GOS [1.8% *w*/*w* of commercial GOS (Oligomate™ 55NP) that contained 55–56% GOS and 44–45% monosaccharides]	A mixture of *S. Typhimurium* FNR-HA—kanamycin-resistant (ATCC 14028s) and *S. Enteritidis* FNR-HA—chloramphenicol-resistant and rifampicin-resistant (ATCC 31194) orally	Increased the level of *Lactobacillales* Decreased the level of *Clostridiales* Reduced colonization of *Salmonella*	[127]
Rhesus monkeys	100 mg/kg/day 3′SL 500 mg/kg/day 3′SL 500 mg/kg/day 3′SL plus omeprazole 500 mg/kg/day 3′SL plus bismuth subsalicylate	Experimentally inoculated with a mixture of 7 *H. pylori* strains (cagA and vacA) isolated from patients	Decreased *H. pylori* colonization in some rhesus monkeys	[40]
Grass carp	0, 200, 400, 600, 800, and 1000 mg/kg MOS	Injections of *A. hydrophila*	Enhanced antimicrobial peptides expression Attenuated inflammatory response Regulated immune barrier function	[130]

Abbreviations: HMOs, human milk oligosaccharides; 2′F, 2′-fucosyllactose; LNnT, lacto-N-neotetraose; FOSs, fructo-oligosaccharides; scFOSs, short-chain fructo-oligosaccharides; lcFOSs, long-chain fructo-oligosaccharides; GOSs, galacto-oligosaccharides; PDX, polydextrose; SCFAs, short-chain fatty acids; PBS, phosphate-buffered saline; Th1, T-helper 1 cells; pAOSs, pectin-derived acidic oligosaccharides; IL, interleukin; TNF-α, tumor necrosis factor alpha; 3′SL, 3′-sialyllactose; 6′SL, 6′-sialyllactose; XOSs, xylo-oligosaccharides; LSTc, lacto-N-triose c; COSs, chitosan oligosaccharides; NLRP3, NOD-like receptor family pyrin domain containing 3; BALF, bronchoalveolar lavage fluid; STAT3, signal transducer and activator of transcription 3; Th17, T-helper 17 cells; MOSs, mannan oligosaccharides; INF-γ, interferon gamma; cagA, cytotoxin-associated gene A; vacA, vacuolating cytotoxin A.

## 3. Antiviral Effects of NDOs

Compared with the described antimicrobial effects of NDOs, limited information on the antiviral effects of NDOs is available. The antiviral effects of NDOs have primarily been studied in the context of gastrointestinal and respiratory viral infections in vivo. NDOs have been shown to synergize with HMOs and exhibit promising antiviral effects [22,23].

### 3.1. In Vivo Evidence for Antiviral Effects of NDOs

In vivo evidence for the antiviral effects of HMOs comes from both animal studies and observational studies in humans. Clinical reports have shown that breastfed infants have a lower incidence of rotavirus (RV), respiratory syncytial virus (RSV), and norovirus than formula fed infants, probably due to the antiviral activities of HMOs [140,141]. RV is a leading cause of severe diarrhea in infants and young children [142]. Studies have shown that feeding neonatal rats with the HMO, 2′-fucosyllactose (2′-FL), alleviates the RV-induced diarrhea by reducing the incidence, duration, and severity of symptoms. The major impact of 2′-FL is observed in early life immune responses like decreased levels of pro-inflammatory cytokines (IL-1β, IL-6, IL-10, IL-12, IFN-γ, TNF-α) and plasma IgA levels in the RV-infected group [23]. Additionally, 2′-FL supplementation was found to increase the expression of toll-like receptors (TLRs) 5 and 7, both of which are important for immune system activation in the RV-infected rats. Furthermore, 2′-FL improved intestinal dysbiosis, a microbial imbalance often seen in RV infections, restoring the gut microbiota toward a healthier state [22]. Dietary supplementation of various HMOs, including 2′-FL, lacto-N-neotetraose (LNT), 6′-sialyllactose (6′-SL), 3′-sialyllactose (3′-SL), and free sialic acid, in RV-infected neonatal piglets significantly elevated the immune cell populations, like memory effector T cells in mesenteric lymph nodes (MLN) and natural killer (NK) cells in peripheral blood mononuclear cells (PBMCs) [26].

Dietary 3′-SL has shown promising effects in improving the clinical signs associated with H9N2 avian influenza (AI), with virus particles undetectable in the swabs of chickens receiving 3′-SL [143]. Similarly, 3′-FL protected mice from a lethal influenza challenge, demonstrating broad activity against multiple influenza strains and even SARS-CoV-2 [144]. The role of HMOs in maintaining gut health, regulating inflammation, and improving overall immunity has generated considerable interest, offering new avenues for therapeutic interventions [36,145,146] and reinforcing the importance of dietary considerations in COVID-related research [147,148,149].

In other studies, a mixture of GOSs (GOSs), long-chain FOSs (lcFOSs), and pectin-derived acidic oligosaccharides (PaOSs) showed protective immunity against RSV, enhancing viral clearance in a formalin-inactivated RSV vaccination model [150]. A similar NDO mixture was tested in suckling rats, where it reduced RV-induced diarrhea, decreased viral shedding, and modified humoral immune responses [28]. Healthy or RV-exposed newborn pigs fed with GOSs and lcFOSs demonstrated higher quantities of immune cell populations including NK cells, MLN effector memory T cells, and basophils compared with the control or RV-infected pigs. However, this immune response was milder compared with pigs fed with an HMO mixture (2′-FL, LNnT, 6′-SL, 3′-SL, and free sialic acid) [26]. Two other studies using a GOS/lcFOS mixture, either alone or combined with 2′-FL, showed reductions in diarrhea severity and RV particles in suckling or neonatal rats [22]. Notably, the NDO mixture, both alone and with 2′-FL, prevented RV-induced gut permeability disruption and the RV-induced increase in the plasma IgA levels [23]. GOS/lcFOS treatment modulated specific anti-RV antibodies, increasing the levels of anti-RV IgA, IgG, and IgM in serum while reducing the anti-RV IgA and IgM levels in the intestine [29].

Three in vivo studies described the antiviral functionalities of MOSs. Porcine reproductive and respiratory syndrome virus (PRRSV)-infected pigs fed with MOSs displayed notable health improvements including an improved gain-to-feed ratio, lower rectal temperatures, and an early increase in white blood cells (WBC). In the late phase of infection, MOSs had the capacity to reduce inflammation by modifying cytokine secretion, like increasing the IL-10 levels [25]. Similarly, in shrimp infected with white spot virus (WSV), one of the most important aquaculture viral diseases [21,151], MOS supplementation resulted in decreased mortality, increased total hemocyte count (THC), and enhanced respiratory burst activity [21]. The antiviral effects of MOSs were further investigated in zebrafish infected with spring viremia of carp virus (SVCV). MOS supplementation improved the survival rates and contributed to a more balanced microbiota composition [27]. Taken together, the in vivo evidence presented above suggests the antiviral potential of NDOs, including HMOs, indicating their possible role in combating viral infections. Table 2 provides an overview of the in vivo evidence for the antiviral effects of NDOs.

### 3.2. Mechanisms of Antiviral Properties of NDOs: Direct Interaction with Pathogens Through Binding Affinity

Although this review primarily focused on in vivo studies, the following section explores potential mechanisms of the antiviral action of NDOs, partly drawing on in vitro experiments that may help deepen our understanding of their in vivo effects.

For the direct antiviral effects of NDOs, the primary mechanism observed was their binding affinity to viral pathogens. To investigate the mechanism of reduced viral shedding observed in RV-infected animals supplemented with the NDO mixture (GOS, lcFOS, and PaOS), Rigo-Adrover and colleagues conducted an in vitro blocking assay. Their findings clearly showed that a GOS/lcFOS or GOS/lcFOS/PaOS combination reduced the RV particles up to 40% [28]. Azagra-Boronat et al. reported a similar in vitro binding affinity for GOS/lcFOS to RV, which was significantly multiplied when 2′-FL was added to these NDOs [23]. However, no specific binding activity was detected for 2′-FL alone [23]. Sialylated HMOs (3′-SL and 6′-SL) were effective in neutralizing different subtypes of AI in vitro, whereas 3′-SL displayed the highest ability in hemagglutination inhibition against all AI subtypes [143]. The sialic acid moiety of these HMOs can bind to hemagglutinin present in the AI virus, preventing hemagglutinin-mediated binding to the host cells [152]. The HMO 3′-SL was also highly effective against AI in vivo, as no particles could be detected in the oral and cloacal swabs taken from 3′-SL-fed chickens [143]. MOSs showed different antiviral activities against different viruses. SVCV viral particle adsorption and replication blockage was clearly shown in the MOS-treated zebrafish ZF4 cells in vitro [27], while no viral load changes were reported in the serum of MOS-fed PRRSV-infected pigs [25]. Notably, the binding capacity of HMOs and NDOs seems to be dependent on the specific viral strain and experimental design.

### 3.3. Mechanisms of Antiviral Properties of NDOs: Indirect Interaction with Pathogens

#### 3.3.1. Effects of NDOs on Intestinal Barrier Function and Intestinal Maturation in Viral Infections

There is evidence that NDOs affect the intestinal maturation process and intestinal barrier function during viral infections. Maturation of the intestinal epithelial barrier in neonatal rats corresponds with decreased FcRn expression [153]. In RV-infected rats, intestinal FcRn expression tended to decrease, while GOS/lcFOS ± 2′-FL supplementation further reduced FcRn expression, suggesting that these compounds may accelerate intestinal maturation [23].

As an indicator for gut permeability, the intestinal A1AT levels were measured in RV-infected suckling rats supplemented with GOS/lcFOS, 2′-FL, or their combination [23]. The A1AT levels were higher in the RV group, while after GOS/lcFOS supplementation, the A1AT levels slightly decreased, regardless of the infection status. The decrease in A1AT levels was more obvious after 2′-FL supplementation compared with scGOS/lcFOS. Moreover, supplementation with GOS/lcFOS, 2′-FL, and their combination effectively decreased the *Muc2* intestinal barrier gene expression, which was increased due to RV infection [23]. GOS/FOS, but not 2′-FL, in the RV-infected suckling rats increased the villi height, width, and area, promoting a mucosa that resembled a healthy, well-differentiated intestine [23,154].

#### 3.3.2. Effects of NDOs on Immune Parameters During Viral Infections

One of the main antipathogenic effects of NDOs is related to the modulation of the host immune responses. However, the exact mechanism underlying these altered immune responses is not yet completely understood, as direct NDO interaction with immune system components [23,155] as well as indirect immunomodulatory effects by modifying the intestinal microflora composition have been observed [22,27].

The immunoglobulin IgA plays a very important role in the first defense against infections [156]. Increased plasma IgA levels in the RV-infected rats were significantly decreased by 2′-FL and GOS/lcFOS supplementation, while combining 2′-FL with GOS/lcFOS reduced the IgA levels even further. The RV-induced elevated plasma levels of IgG2b were reduced in both the GOS/lcFOS- and 2′-FL-supplemented animals (and in GOS/lcFOS + 2′FL group), while the IgG1 and IgG2c levels were only decreased by GOS/lcFOS supplementation [23]. Rigo-Adrover and colleagues reported a significant increase in the anti-RV IgA, IgG, and IgM levels in the serum of infected animals supplemented with GOS/lcFOS. In the intestinal washes of these animals, the IgM and IgA levels were reduced, while IgA increased in the early stage of the infection [29]. These high anti-RV IgA, IgG, and IgM levels in the systemic circulation induced by scGOS/lcFOS seem to indicate protection against the RV infection [157]. The early increase in IgA levels in the intestinal washes by GOS/lcFOS could be due to a higher binding to RV, leading to higher mucosal clearance to inhibit infection [24,158]. In addition, increased levels of fecal IgA have been reported in infants receiving scGOS/lcFOS [24].

The immunomodulatory potency of 2′-FL was demonstrated by an inhibition in the RV-induced increase in intestinal cytokine levels including IL-1β, IL-6, IL-10, IL-12, IFN-γ, and TNF-α [23]. The immunomodulatory effects of 2′-FL might be microbiota-dependent, but can also be the result of the direct linkage of 2′-FL to immune cells by mimicking selectin ligands [159].

The effect of NDOs on host immune responses during viral infections has also been investigated, focusing on Th1 and Th2 responses [23,150]. GOS/lcFOS with or without PaOS altered the Th1/Th2 ratio during RSV and RV infections. Schijf et al. reported an increased Th1 response and a reduced Th2 response by measuring RSV-specific Th2 cytokine (IL-4, IL-5, and IL-13)-producing CD4^+^ T cells, induced by GOS/lcFOS/pAOS [150]. Azagra-Boronat et al. showed that all tested NDOs (GOS/lcFOS/2′-FL) effectively lowered the Th1/Th2 levels, which typically rise during RV infection. Specifically, 2′-FL showed the highest reduction in Th1/Th2 by lowering the Th1 indicator (IgG2b), while GOS/lcFOS simultaneously decreased both the Th1 and Th2 (IgG1) indicators. The combined treatment of GOS/lcFOS and 2′-FL exhibited an average level between the levels of individual treatments [23].

It has also been reported that NDOs, like MOSs, can alter immune responses in pigs infected with PRRSV. Dietary MOSs are related to a rapid increase in WBC numbers during the early stage of PRRSV infection, while the anti-inflammatory properties of MOSs, associated with lower serum TNF-α levels and higher IL-10 release, were observed at the end of the acute phase of PRRSV [25]. MOSs also had the capacity to boost host immune responses against viral infections like SVCV by increasing the expression of the type I IFN signaling pathway genes (e.g., IFNφ1, IFNφ2, IFNφ3) in zebrafish ZF4 cells in vitro [27].

The HMO 3′-FL is also recognized as an immunomodulator that helps in protecting the host from viral infections, as evidenced by enhanced leukocyte migration and reduced viral titers in influenza-infected mice. During viral infections, 3′-FL induced elevated levels of interferon receptors, which promoted antiviral innate immunity. This included the production of nitric oxide production, the expression of interferon-stimulated genes, and the activation of genes related to innate immune cells that have the capacity to inhibit viral infections [144].

Finally, NDOs might also be responsible for altered TLR signaling during viral infections. TLRs are a family of pattern recognition receptors that are crucial in the first line of defense against pathogens, like viruses [160]. NDOs can influence the immune system through their effects on the gut microbiota, which in turn can affect the activation of virus-recognizing TLRs [4,5,6,17,18,161]. GOS/lcFOS has been shown to upregulate the transcription of TLR9 (recognizing unmethylated DNA) in RV-infected rats, while 2′-FL alone and in combination with GOS/lcFOS upregulated the transcription of TLR7 (recognizing single-stranded RNA) [22]. The NDOs, FOSs, GOSs, and MOSs, have been shown to activate TLR4 (recognizing LPS and viral glycoproteins) [162], while LNT2 could activate all TLRs [163]. Hence, the mentioned NDOs play a role in modulating the immune response through various mechanisms like modifying the levels of immunoglobulins at different stages of viral infections, mitigating the excessive release [17,161] of cytokines in response to viral infections, influencing WBC counts, stimulating TLRs, and producing immunostimulatory by-products via degrading virus particles.

#### 3.3.3. Effects of NDOs on Microbiota Composition During Viral Infections

The findings illustrate the beneficial effects of GOS/lcFOS, 2′-FL, and their combination on RV-induced intestinal dysbiosis. Specifically, the *Streptococcaceae* and *Staphylococcaceae* bacterial amounts were reduced in the RV group compared with the control group, which was prevented by supplementation with GOS/lcFOS, 2′-FL, and their combination [22].

Investigation of the gut microbiota composition in SVCV-infected zebrafish revealed distinct changes in bacterial populations induced by MOSs, favoring the growth of Proteobacteria. Conversely, dietary MOSs led to a reduction in the population of Firmicutes and Actinobacteria [27].

The interaction between microbiota and viral infections is not fully understood, and more information needs to be gathered on how intestinal microbiota can boost the host’s immune response, regulate viral infections, and improve host immunity, specifically focusing on antiviral defenses [164,165,166].

#### 3.3.4. Effects of NDOs on SCFA Production During Viral Infections

SCFAs are crucial for protecting the intestinal barrier and regulating the immune response during infections. SCFAs have been shown to have the opposite effects on viral pathogens, either promoting or inhibiting viral replication, depending on the type of virus at hand [145]. Most NDOs are metabolized into SCFAs by the intestinal microbiota [146]. Recently, it has been shown in mice that acetate and butyrate protect against pulmonary viral infection by maintaining barrier integrity and immunity against viral infections [145,167]. However, butyrate treatment has been shown to increase histone acetylation, which increases the transcription of viral genes in latently infected epithelial cells, ultimately leading to virus reactivation [168]. Furthermore, butyrate increases cellular infection with the influenza virus, reovirus, HIV-1, human metapneumovirus, and vesicular stomatitis virus [169]. The HMO 2′-FL made no significant changes on the cecal SCFA in RV-infected suckling rats [23]. Remarkably, when 2′-FL together with prebiotic GOS/lcFOS was given, all of the SCFA levels were reduced compared with the rats only exposed to RV [23]. However, in another study with a comparable experimental design, no discernible changes in the cecal SCFA levels following the administration of GOS/lcFOS were observed [29]. Future research should continue to explore the precise mechanisms by which NDOs and SCFAs interact with the immune system and viral pathogens, potentially leading to new therapeutic strategies for managing viral infections.

**Figure 3:**
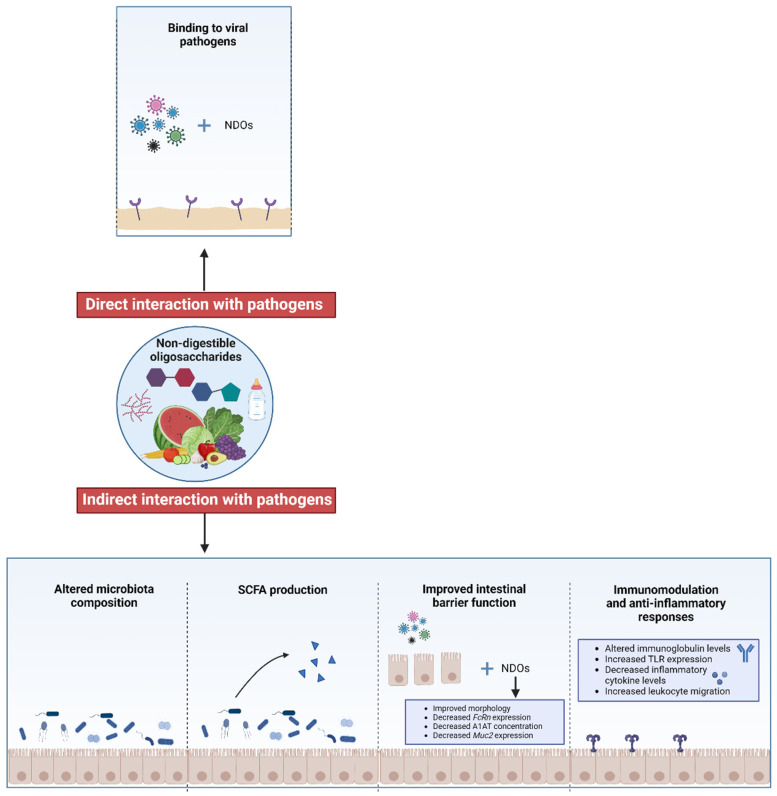
Hypothesis for the mechanism of action of non-digestible oligosaccharides (NDOs) against viral infections. This illustration outlines the key mechanisms through which NDOs exert their antiviral effects, both directly and indirectly, via interactions with pathogenic bacteria. Firstly, NDOs can directly bind to specific viral pathogens, potentially neutralizing their activity [23,27,28,143]. Indirectly, NDOs modulate the intestinal microbiota composition [22,27], leading to increased levels of short-chain fatty acids (SCFAs) [23,168], which can further inhibit viral infection. Additionally, NDOs enhance intestinal barrier function (e.g., by improving gut morphology, reducing the *FcRn* and *Muc2* RNA expression levels, and inhibiting A1AT levels) [23,145,154]. Moreover, NDOs function as immunomodulators and anti-inflammatory agents by altering the immunoglobulin levels, increasing TLR expression, reducing the inflammatory cytokine levels, and enhancing leukocyte migration [22,23,25,26,27,28,29,144,150]. Created with BioRender.com (accessed on 16 February 2025). NDOs, non-digestible oligosaccharides; SCFAs, short-chain fatty acids; FcRn, neonatal Fc receptor; A1AT, alpha-1 antitrypsin; Muc2, mucin 2; TLR, toll-like receptor.

Overall, NDOs can mitigate virulence by binding to specific viruses. Additionally, NDOs promote a healthier gut microbiota composition, leading to increased SCFA levels and the suppression of viral replication. NDOs also enhance intestinal barrier function, potentially by regulating impaired intestinal immune homeostasis or modulating genes associated with barrier integrity. Figure 3 illustrates the proposed mechanisms of action of NDOs against viral infections.

**Table 2:** In vivo evidence for the antiviral effects of NDOs.

Treatment Target	NDOs (Amount, Application, etc.)	Model Description (Pathogen, Application, etc.)	Effects	References
Neonatal piglets	2′-FL, LNnT, 6′-SL, 3′-SL, and free sialic acid (4 g/L), scGOS/lcFOS (3.6 g + 0.4 g) per liter	RV (OSU: Ohio State University)	Increased systemic and gastrointestinal immune cells	[26]
Suckling rats	2′-FL (0.2 g/100 g BW), scGOS/lcFOS (9:1) 0.8 g/100 g of BW and 2′-FL combined with scGOS/lcFOS (0.2 + 0.8 g/100 g BW)	RV (simian SA-11) orally inoculated at day 5 of life	Ameliorated RV-induced clinical symptoms Decreased intestinal cytokines levels Altered immunoglobulin levels (reduced IgA levels) Decreased viral shedding and high RV binding activity Decreased A1AT concentration and *MUC2* gene expression Decreased FcRn gene expression levels Improved morphological status of the gut Reduced cecal SCFA levels Decreased Th1/Th2 ratio	[23]
Neonatal rats	2′-FL (0.2 g/100 g BW), scGOS/lcFOS (9:1) 0.8 g/100 g of BW and 2′-FL combined with scGOS/lcFOS (0.2 + 0.8 g/100 g BW)	RV (simian SA-11) orally inoculated at day 5 of life	Ameliorated RV-induced clinical symptoms Elevated TLR5, TLR7 and TLR9 gene expression levels Inhibited RV-induced dysbiosis Increased viral clearance	[22]
Chickens	3′-SL (1 mL of 500 mM 3′-SL per day)	AI (H9N2)	Ameliorated clinical symptoms Removed virus particles from cloacal and oral swabs	[143]
Piglets	2 mg/mL of LNnT, acidic HMO mixture (40% 6′-SL/10% 3′-SL/50% SA) Directly injected into ileal loops for 6 h	RV (OSU: Ohio State University)	Decreased RV replication in ileal loops	[170]
*C57BL/6* mouse	ScGOS/lcFOS/pAOS (9:1:10) 2% (*w*/*w*) of the total carbohydrate in the diet	RSV strain A2 (VR-1302; ATCC) and (FI)-RSV vaccine	Increased viral clearance Increased Th1 responses	[150]
Suckling rats	scGOS/lcFOS/pAOS (7.6:8.5:15) 0.8 g/100 g of BW	RV (simian SA-11) orally inoculated at day 7 of life	Ameliorated RV-induced clinical symptoms. Increased viral clearance. Increased anti-RV antibodies in serum	[28]
Suckling rats	scGOS/lcFOS (9:1) 0.8 g/100 g of BW	RV (simian SA-11) intragastrically inoculated at day 7 of life	Increased anti-RV antibodies in serum Decreased anti-RV antibodies in intestine (except IgA) No effects on SCFAs	[29]
Nursery pigs	MOS 0.2% (Bio-Mos, Alltech Inc., Nicholasville, KY, USA)	PRRSV (Purdue isolate P-129) intranasally inoculated at week 5 of life	Ameliorated PRRSV-related clinical symptoms. Decreased inflammatory cytokine levels and increased IL-10 levels in serum Increased WBC levels No effect on viral clearance	[25]
Mice	2′-FL, 3′-FL and 3′-FL + 2′-FL (first study: 750 mg/kg, second study: 150 mg/kg), 100 µL/oral gavage	Intranasal infection with H1N1 (A/Puerto Rico/8/34)	Decreased lethality Decreased viral titers Increased leukocyte migration	[144]
Tiger shrimp *(Penaeus mondon*)	MOS + peptidoglycan (0.1, 0.2 and 0.4%)	WSV-infected water for one hour	Decreased mortality ratio Increased immune indicators (total hemocyte count and respiratory burst activity)	[21]
Tuebingen zebrafish	MOSs (0.2, 0.4, 0.6, and 0.8%)	SVCV bath immersion for 12 days	Increased survival rate Modified intestinal microflora composition Increased viral clearance Increased expression of type I IFN signaling pathway genes	[27]

Abbreviations: 2′-FL, 2-fucosyllactose; 3′-FL, 3-fucosyllactose; LNnT, lacto-N-neotetraose; 6′-SL, 6-sialyllactose; 3′-SL, 3-sialyllactose; RV, rotavirus; scGOSs, short-chain galacto-oligosaccharides; lcFOSs, long-chain fructo-oligosaccharides; IgA, immunoglobulin A; BW, body weight; Muc2, mucin 2; A1AT, alpha-1 antitrypsin; FcRN, neonatal Fc receptor; TLR, toll-like receptor; AI, avian influenza; LnNT, lacto-N-tetraose; HMOs, human milk oligosaccharides; pAOSs, pectin-derived acidic oligosaccharides; RSV, respiratory syncytial virus; Th1, T-helper 1 cells; IL-10, interleukin 10; MOSs, mannan oligosaccharides; PRRSV, porcine reproductive and respiratory syndrome virus; WSV, white spot virus; SVCV, spring viremia of carp virus; WBC, white blood cell count; IFN, interferon.

## 4. Conclusions and Future Prospective

This review compiled current in vivo evidence supporting the antimicrobial and antiviral properties of NDOs, exploring the potential mechanisms of action that encompass both direct and indirect pathways. NDOs, including HMOs, demonstrate antipathogenic effects primarily by directly binding to pathogens, disrupting their virulence, and hindering host cell invasion. These interactions trigger downstream biological responses that contribute to the mitigation of pathogen-induced damage. Additionally, indirect pathways, such as the modulation of gut microbiota and the enhancement of immune responses, play a crucial role in supporting the host’s defense against infections.

While promising, further research is needed to fully explore the antibacterial and antiviral spectrum of NDOs and their role as therapeutic alternatives to traditional anti-infective drugs. Current gaps in knowledge include the identification of NDOs suitable for targeting a range of bacterial species, particularly those with differing cell wall structures (Gram-positive and Gram-negative). To gain a deeper understanding of their broad-spectrum efficacy, future studies should include a wider variety of bacterial and viral species. A better understanding of the structural characteristics and physicochemical properties of NDOs, such as electrical charge, degree of polymerization (DP) length, pH stability, and other key attribute, will provide insights into their mechanisms of action, elucidate the structure–function relationship, and improve their application as anti-infective agents. Importantly, a combination of structurally diverse NDOs may be required to optimize anti-infective effects, suggesting that tailored blends can be particularly effective in maintaining gut health and immune balance in the face of complex multi-pathogen challenges. While it might be challenging to fully replace antimicrobials with NDOs, their potential to enhance the efficacy of conventional antimicrobials is an exciting area of research. This approach holds significant implications for public health strategies aimed at combating antimicrobial resistance. Future research should focus on clinical trials and mechanistic studies to confirm the synergistic effects of NDOs and conventional antimicrobials and to better understand their interactions at the molecular level. Additionally, while the modulation of gut microbiota composition is an established indirect pathway for NDOs to exert their anti-infective effects, it is important to consider individual variations in gut microbiota. This variability underscores the need for personalized approaches in NDO administration to maximize the therapeutic outcomes. Despite intriguing findings regarding the potential benefits of NDOs against respiratory viruses like RSV, influenza, and COVID-19, more research is needed in this area. The strict safety requirements for viral studies may partly explain the limited data, but it is essential to prioritize the initiation of large-scale clinical trials to validate these effects and address potential future viral outbreaks.

In summary, the evidence outlined in this review supports the anti-infective potential of NDOs through both direct interactions with pathogens and the modulation of gut microbiota. NDOs can neutralize pathogen virulence, inhibit growth, reduce pathogen–host cell interactions, and enhance the efficacy of antibiotics. Moreover, they contribute to a healthier intestinal environment by fostering beneficial microbiota, increasing SCFA production, supporting immune homeostasis, and strengthening the intestinal barrier. Given the increasing relevance of glycobiology, NDOs hold promise as a new class of anti-infective agents that combine the benefits of both food and drug applications. However, their transition to therapeutic use will require more clinical evidence to overcome the current limitations and explore their potential as complementary agents or alternatives to conventional antimicrobials.
